# A fronto-insular network underlies individual variations in anger expression and control

**DOI:** 10.1162/imag_a_00348

**Published:** 2024-11-05

**Authors:** Alessandro Grecucci, Francesca Graci, Ellyson Munari, Xiaoping Yi, Gerardo Salvato, Irene Messina

**Affiliations:** Department of Psychology and Cognitive Sciences, University of Trento, Trento, Italy; Center for Medical Sciences, University of Trento, Trento, Italy; Department of Radiology, Xiangya Hospital, Central South University, Changsha, Hunan, P.R. China; Department of Brain and Behavioral Sciences, University of Pavia, Pavia, Italy; Cognitive Neuropsychology Centre, ASST Grande Ospedale Metropolitano Niguarda, Milan, Italy; NeuroMi, Milan Center for Neuroscience, Milan, Italy; Faculty of Social Sciences and Communication, Universitas Mercatorum, Rome, Italy

**Keywords:** anger externalization, anger control, affective neuroscience, data fusion, unsupervised machine learning

## Abstract

Anger can be deconstructed into distinct components: a tendency to outwardlyexpress it (anger-out) and the capability to manage it (anger control). Theseaspects exhibit individual differences that vary across a continuum. Notably,the capacity to express and control anger is of great importance to modulate ourreactions in interpersonal situations. The aim of this study was to test thehypothesis that anger expression and control are negatively correlated and thatboth can be decoded by the same patterns of grey and white matter features of afronto-temporal brain network. To this aim, a data fusion unsupervised machinelearning technique, known as transposed Independent Vector Analysis (tIVA), wasused to decompose the brain into covarying GM–WM networks and thenbackward regression was used to predict both anger expression and control from asample of 212 healthy subjects. Confirming our hypothesis, results showed thatanger control and anger expression are negatively correlated, the moreindividuals control anger, the less they externalize it. At the neural level,individual differences in anger expression and control can be predicted by thesame GM–WM network. As expected, this network included lateral and medialfrontal regions, the insula, temporal regions, and the precuneus. The higher theconcentration of GM–WM in this brain network, the higher the level ofexternalization of anger, and the lower the anger control. These results expandprevious findings regarding the neural bases of anger by showing that individualdifferences in anger control and expression can be predicted by morphometricfeatures.

## 1 Introduction

Anger is a primary emotion typically characterized by discomfort serving to mobilizeresources and enact change in response to provocation, hurt, or threat (Sorella et al., 2021,^2022^;Videbeck,2006). Its constructive role encompasses facilitating goal achievements,overcoming obstacles, and maintaining interpersonal boundaries (Grecucci, Giorgetta, Brambilla, et al., 2013;Grecucci et al., 2013b;Sorella et al., 2021,^2022^). Nonetheless, anger regulation is challenging due to the intensephysiological reactions associated with the fight-or-flight response, activated tosafeguard oneself from the provoking circumstances (Lazarus, 1991). Difficulties in regulating anger may serve as precursorsof aggressive behaviours (Lochman et al.,2010), with consequent interpersonal difficulties and socialmaladaptation (Baron et al., 2006;Heilbron & Prinstein, 2008). And angerdysregulation is a core feature of psychiatric disorders such as borderlinepersonality disorder (De Panfilis et al.,2019), antisocial personality disorder (Kolla et al., 2017), and intermittent explosive disorder (Coccaro et al., 2014). Therefore, acomprehensive understanding of anger regulation is imperative. A widely recognizedpsychological framework aimed at elucidating the nature and components of angerregulation has been proposed by Spielberger and operationalized in the State andTrait Anger Expression Inventory (STAXI-2;Spielberger, 1999). This model recognizes anger expression and angercontrol as two main dimensions of anger regulation. Anger externalization (AngerExpression—Out) refers to an individual’s propensity to externalize oropenly express their anger, through questions involving behaviours, actions, orreactions in anger-inducing situations, in contrast to anger internalization (AngerExpression—In) that regards the tendency of individuals to suppress orinternalize anger. Anger control refers to people’s ability to control thephysical or verbal expressions of anger (Anger Control-In) and to relax, calm down,and reduce angry feelings before they get out of control (Anger Control—Out).Among such dimensions of anger regulation, externalization and control representindependent but complementary components of anger regulation. Indeed, anger controlcan be viewed as the ability to restrain anger expression through control andoutcome monitoring (Wilkowski & Robinson,2008,^2010^). If on the one handexternalizing anger in same cases may play a positive role (e.g., maintenance ofboundaries) (Grecucci, Giorgetta, Brambilla, et al.,2013;Grecucci et al., 2013b;Sorella et al., 2021,^2022^), on the other hand, when not counterbalanced byadequate anger control may have negative consequences. For instance, the combinationbetween anger externalization and low anger control garners attention because bothare considered precursors of hostility (Bridewell& Chang, 1997) and related social interaction issues (Baron et al., 2006;Messina et al., 2023) and aggressive behaviours (Birkley & Eckhardt, 2015;Lochman et al., 2010;Roberton et al., 2015). Similarly, it has been shown thatpeople with poor control of anger have a greater propensity to angryexternalization, fostering aggressive behaviours (Bettencourt et al., 2006;Dodge &Coie, 1987;Mattevi et al., 2019).From a clinical perspective, these dimensions of anger regulation require tailoredinterventions focused on externalizing psychological problems (distinct from thosetargeting internalized anger expression, more typical of internalizing problems)(Achenbach et al., 2016;Bjureberg et al., 2023;Manfredi & Taglietti, 2022;Pascual-Leone et al., 2013). Despite the importance of individualvariances in regulating anger for mental well-being, there exists limited evidenceregarding the neural foundations of these individual differences.

Anger control involves the ability to manage, calm, and monitor the outcomes ofanger. The Integrative Cognitive Model (Wilkowski& Robinson, 2010) examines the link between trait anger and thecapacity for anger control. Although the role of specific brain areas in angerregulation requires further investigation, the involvement of the prefrontal cortexhas been proposed in a high-road model adapted from fear studies. According toAlia-Klein et al. (2020), uncontrollable angertriggers a low-road brain activation, where higher-order cognition (i.e., theprefrontal cortex) plays a minimal role, leading to aggression with greaterautomaticity. Conversely, higher-order cognition is crucial in regulating anger; forinstance, regulation through reappraisal (an emotion regulation strategy thatchanges the interpretation of a situation) involves the activation of prefrontalregions, such as the inferior frontal gyrus (Grecucci et al., 2013a,2013b).Stimulation of the medial prefrontal cortex can reduce anger and aggression (Gilam et al., 2018). Both medial and lateralprefrontal areas are part of the default mode network, linked to anger control(Sorella et al., 2022) andinternalization (Grecucci et al., 2022). Thisaligns with the neural model of emotion regulation, where implicit regulationinvolves the medial prefrontal cortex, while explicit regulation also engages thelateral prefrontal and parietal cortices (Etkin etal., 2015). Thus, anger may be automatically managed by the medialprefrontal cortex, while conscious regulation involves the right inferior frontalgyrus and other lateral prefrontal regions, especially during strategies such asreappraisal (Grecucci et al., 2013a,2013b). Beside the prefrontal cortex, otherregions may play a role in anger control. At a structural level,Sorella et al. (2021)found that the concentration ofgrey matter in a network comprising ventromedial temporal areas, posteriorcingulate, fusiform gyrus, and cerebellum correlated with trait anger. From anetwork perspective, previous functional studies have also found altered restingstate activity (Sorella et al., 2021) andfunctional connectivity (Fulwiler et al.,2012) in the Default Mode Network (DMN), which includes frontal andtemporal areas, confirming previous observations. Last but not the least, an effortto control anger may increase the connectivity between the amygdala and prefrontalcortices, which are responsible for top–down control (Denson et al., 2013). Accordingly,Fulwiler et al. (2012)showed a positive correlationbetween anger control and the functional connectivity (FC) of the amygdala with thecontralateral orbitofrontal cortex. In line with this finding, it has been reportedthat violent offenders’ resting state activity after being provoked intoanger showed increased amygdala–paralimbic connectivity and decreasedamygdala–medial prefrontal cortex (mPFC) connectivity, suggesting that aninability of regulation inside the mPFC can lead to reactive aggression (Siep et al., 2018). The amygdala andprefrontal cortices, which oversee top–down behaviour, become more connectedwhen individuals make an effort to control their anger in response to an insult(which is simulated in experimental settings using anger provocation paradigms)(Siep et al., 2018). Anger managementand the functional connectivity (FC) of the amygdala with the oblique orbitofrontalcortex were found to be positively correlated (Fulwiler et al., 2012).

More related to externalization, some studies tried to understand the neural bases ofaggression, a dysregulated form of externalization. Researchers found activations infrontal regions, in the insula, and in the striatum to be related with aggressiontendencies (Dambacher et al., 2015;Repple et al., 2018;Skibsted et al., 2017). In another study, aggression wasrelated with both the left superior frontal gyrus and the left middle temporal gyrus(Gong et al., 2022). Additionally, traitimpulsivity that may be related with externalizing anger was linked to prefrontal,temporal, and parietal cortices (Pan et al.,2021). From a structural point of view, a recent study used supervisedmachine learning to identify grey matter features related to the individualdifferences in externalizing anger (Consolini etal., 2022), revealing that the medial, lateral, and orbitofrontalregions, the temporal and parietal regions (temporal poles, insula, fusiform andangular gyrus, posterior cingulate), the basal ganglia, and parts of the cerebellumwere found to be involved in the structural network that predicted angerexternalization.

## 2 Aims of the Study

Thus, the first aim of the present study is to test the hypothesis that there is anegative relationship between the expression and control of anger. We predict thatthis relationship stands true: the more the individuals can control anger, the lessthey externalize it. If this hypothesis is true (negative relationship between angercontrol and anger expression), someone can expect that the same neural circuit isinvolved in both externalizing and controlling anger.

The second aim of this study is to test the hypothesis that the same GM–WMcircuit related to anger externalization may be related with anger control. Toinvestigate the existence of a common neural circuitry for “angerexpression” and “control,” we employed for the first time adata fusion unsupervised machine learning approach known as tIVA to decompose thebrain into covarying GM–WM neural networks, and a Backward Regressionapproach to predict expression (STAXI Anger-outward) and anger control (STAXIAnger-control). In line with the previous evidence on these topics, we expect thatan affective network including subcortical structures such as the amygdala, thebasal ganglia, as well as the frontal network and temporal cortices may be includedin this network. We also expect the cerebellum in having a role on this network. Thecerebellum has been linked to many cognitive and affective functions (Sorella et al., 2022). Finally, we expect thatWM regions related to the connections between these areas should be in the same wayrelated to anger externalization and control.

## 3 Material and Methods

### 3.1 Sample

Behavioural and structural MRI data from a cohort of 212 healthy participants(mean age: 26.06 ± 4.14 years, 131 M, 81 F) were obtained from theMPI-Leipzig Mind Brain-Body dataset available at OpenNeuro Dataset,http://openneuro.org,RRID:SCR_005031, accession number ds000221 (Babayan et al., 2020). This project was approved by the ethicscommittee of the University of Leipzig (Ethics Committee Approval Number:097/15-ff). Participants were selected for the project with the followingexclusion criteria: pregnancy, metallic implants, braces, non-removablepiercings, tattoos, claustrophobia or tinnitus, and surgical operation in thepast 3 months. Individuals with any history of psychiatric diseases thatrequired inpatient treatment for longer than 2 weeks within the past 10 years,or history of neurological disorders (including multiple sclerosis, stroke,epilepsy, brain tumour, meningoencephalitis, severe concussion) were excludedtoo. Moreover, individuals with intake of active drugs, beta- and alpha-blocker,cortisol, and any chemotherapeutic or psychopharmacological medication wereexcluded. Finally, individuals with positive drug anamnesis (extensive alcohol,MDMA, amphetamines, cocaine, opiates, benzodiazepine, cannabis) were excludedtoo. They also met the MRI safety requirements of the MPI-CBS (Mendes et al., 2019) and provided informed consentbefore the experimental sessions. The final sample was determined based onspecific criteria, including the availability of structural images, ages rangingfrom 20 to 45 years, and the availability of scores from STAXI. This age rangewas selected to exclude potential effect of ageing on brain networks. Previousstudies have shown that GM and WM concentrations inside macro networks detectedby ICA- and IVA-based approaches are subjected to effect age (see, e.g.,Baggio et al., 2023). Adding individualsoutside our age range would have added noise to the analyses.

### 3.2 Questionnaire data

The State and Trait Anger Expression Inventory-2 (STAXI-2;Spielberger, 1988) was considered to assess angerfacets. In line with the aims of the present study, and to test the hypothesisof a negative relationship between externalization and control, and that theyrely on the same brain circuit, we considered participants’ score on thesubscale Anger-Expression-Out that refers to the extent to which people expresstheir anger outwardly in a poorly controlled manner (i.e., the externalizationof anger; eight items, e.g., “I do things like slam doors”). Wealso considered scores on the Anger-Control subscales, which assessindividuals’ ability to monitor and control their emotions, by calmingdown (Anger-Control-In; 8 items, e.g., “I control my angryfeelings”) and avoiding anger externalization through physical and verbalexpressions (Anger-Control-Out; eight items, e.g., “I control mybehaviour”). The STAXI scales have demonstrated adequate convergent anddiscriminant validity (Deffenbacher et al.,1996), internal consistency (Fuquaet al., 1991;Spielberger,1996), test–retest reliability (Jacobs, Latham, & Brown, 1988), and a stablefactor structure (Forgays, Forgays, &Spielberger, 1997;Fuqua et al.,1991). Indeed, the STAXI has been defined as an instrument withstrong psychometric properties (Foley et al.,2002). Preliminary research has also supported the validity of themore recently developed Anger-Control subscale (Spielberger et al., 1985). In a more recent validation, the STAXI-2scales showed to be reliable in terms of both internal consistency andtest–retest reliability. Indeed, the internal consistency of the STAXI-2was adequate, with alpha coefficients for the STAXI-2 scales all above 0.70, anda test–retest reliabilities fairly stable (Lievaart et al., 2016). Of note, the STAXI-2 used inthis study was the German validated questionnaire that proved the goodness ofthe original scale (Tibubos et al.,2020). In the present study, participants had a mean of 11.929 (SD= 3.33) for anger externalization and of 22.726 (SD = 3.78) foranger control.

### 3.3 MRI data

The MPI-Leipzig Mind Brain-Body dataset comprises anatomical, functional, andresting-state data collected at the Day Clinic for Cognitive Neurology,University of Leipzig, utilizing a 3 T Siemens Magnetom Verio scanner (MagnetomVerio, Siemens Healthcare, Erlangen, Germany) equipped with a 32-channel Siemenshead coil. Each participant underwent a comprehensive imaging protocol, whichincluded a high-resolution structural scan, four resting-state fMRI scans, twogradient echo field maps, two pairs of spin echo images with reversed phaseencoding direction, and a low-resolution structural image acquired using a FluidAttenuated Inversion Recovery (FLAIR) sequence, typically employed in clinicalprotocols (Mendes et al., 2019). For thepurposes of our research, we exclusively utilized the structural imagesavailable within the MPI-Leipzig Mind Brain-Body database. These structuralimages were acquired using the MP2 RAGE sequence (Marques et al., 2010) and were characterized by thefollowing parameters: TR = 5,000 ms; TE = 2.92 ms; TI1 =700 ms; TI2 = 2,500 ms; flip angle 1 = 4°; flip angle 2= 5°; voxel size = 1.0 mm isotropic; FOV = 256× 240 × 176 mm; bandwidth = 240 Hz/Px; GRAPPA accelerationwith an iPAT factor of 3 (32 reference lines); prescan normalization; and atotal acquisition time of 8.22 minutes.

### 3.4 Preprocessing

All structural MRIs (sMRI) underwent preprocessing using SPM12 (SPM,https://www.fil.ion.ucl.ac.uk/spm/software/spm12/, RRID:SCR_007037).Initially, we conducted a comprehensive data quality check to identify andaddress potential distortions, including issues such as head motion orartefacts. Subsequently, we executed the reorientation procedure to align theimages according to a common reference point and conducted image segmentation todelineate grey matter, white matter, and cerebrospinal fluid using the CAT12toolbox (Computational Anatomy Toolbox for SPM,http://www.neuro.uni-jena.de/cat/, RRID:SCR_019184). For the currentresearch, both grey and white matter images were utilized in our analyses. Tofacilitate registration, we employed the Diffeomorphic Anatomical Registrationusing Exponential Lie algebra (DARTEL) tools for SPM12 (https://github.com/scanUCLA/spm12-dartel). Finally, we conductedimage normalization to the MNI (Montreal Neurological Institute) space, followedby spatial smoothing using a Gaussian kernel with a full width at half maximum(FWHM) of 12 following the suggestions byChenand Calhoun (2018).

### 3.5 Machine learning analyses

To investigate the neural underpinnings of the anger constructs of interest, weemployed the unsupervised machine learning method known as TransposedIndependent Vector Analysis (tIVA) (Adali et al.,2015). Transposed Independent Vector Analysis (tIVA) is a blindsource separation method (BSS), which provides a fully multivariate approach andenables fusion of data from multiple modalities, such as GM and WM and thendecomposing the brain into joint GM–WM profiles (Adali et al., 2015). The tIVA method is an extensionof Independent Component Analysis (ICA) that exports statistical independenceand generalizes ICA to multiple datasets by analyzing data across datasets,enabling multimodal fusion. tIVA was applied to structural data by using theFusion ICA Toolbox (FIT,http://mialab.mrn.org/software/fit) (Calhoun & Adali, 2006) in the MATLAB 2018a environment (https://it.mathworks.com/products/matlab.html) (*MATLAB(R2018a)*). The number of components for both modalities wasestimated via the minimum norm criterion. To investigate the reliability of eachmodality, the ICASSO (Himberg &Hyvarinen, 2003;Himberg, Hyvarinen,& Esposito, 2004) and the Infomax algorithms were used. ICASSOrepeats the analysis multiple times with different random initializations andsubsequently quantifies the consistency of the outcomes (Wei et al., 2022). The following parameters wereselected: one group with 212 subjects, 2 features (GM and WM images), number ofcomponents 8, normalization in z-scores, RandInit mode (randomizing differentinitial values), PCA standard type, default mask, ICA options: learning rate= 0.0072, max steps = 512, annealing 0.9000, annealdeg =60, posact=on, sphering=off, bias=on, verbose=on.The resulting output consisted of a matrix with the number of subjects (rows)and the loading coefficients for each component (columns). Loading coefficientsrepresent how each component is expressed for every participant in terms of GMand WM concentration (density of values in each voxel). Subsequently, theindependent components were translated into Talairach coordinates. Finally, thesignificant networks were plotted in Surf Ice (https://www.nitrc.org/projects/surfice/).

## 4 Results

### 4.1 Behavioural result

**Fig. 1.**
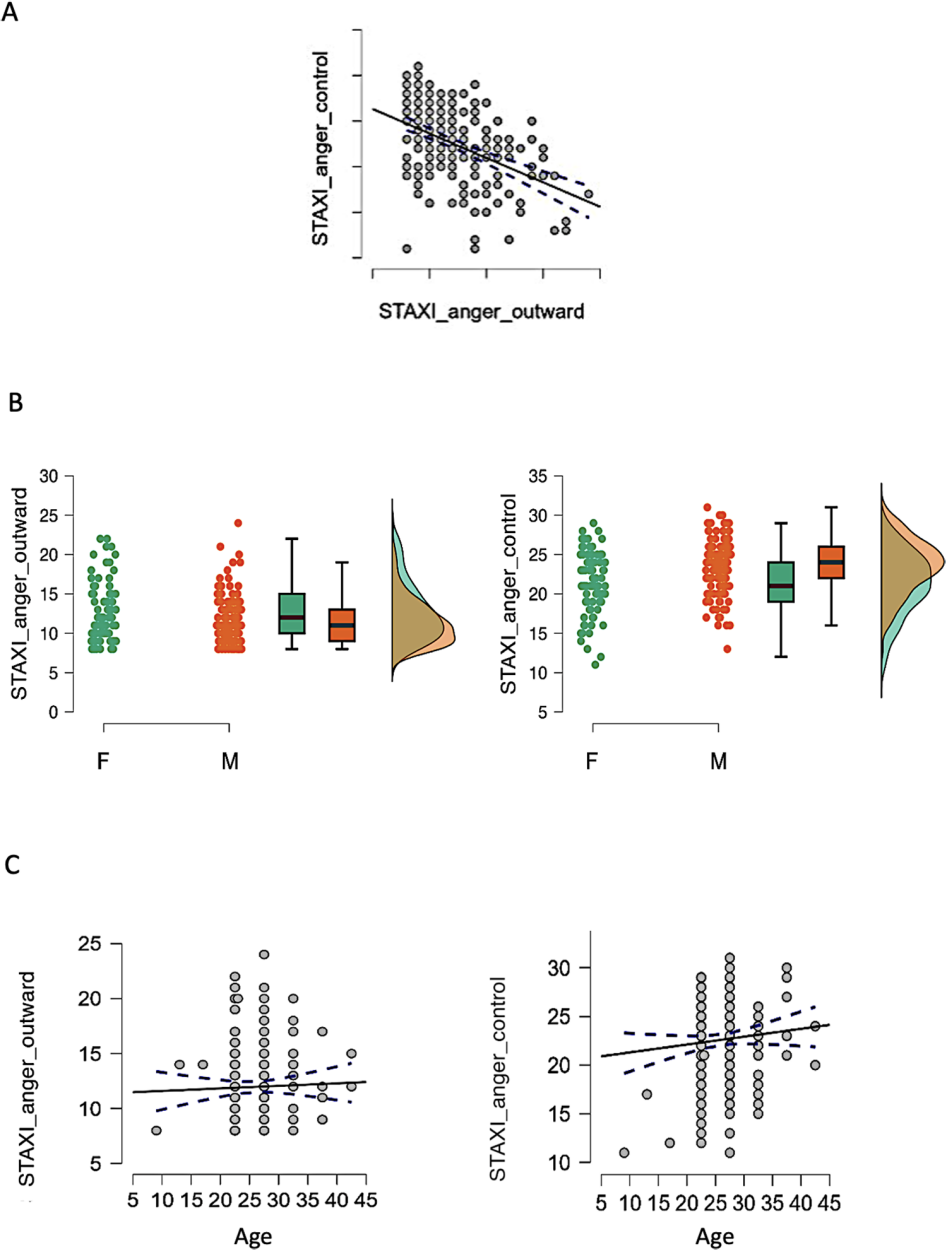
Behavioural results. (A) Negative correlation between anger control andanger externalization. (B) t-Tests for gender effects in angerexternalization and anger control. (C) Correlations between angerexternalization, anger control, and age.

To explore the relationship between anger externalization and control, weperformed a correlation analysis, which revealed a significant negativeassociation between the two scales (rho = -0.418, p = 0.001;Fig. 1A). To assess eventual effects ofgender, we conducted two sample t-tests to explore the potential influence ofgender on anger externalization (t = 2.968; p = 0.003) and angercontrol (t = -4.436; p = 0.001) which confirmed genderdifferences. Additionally, we performed a correlation analysis to assess theimpact of age on both anger externalization and control. Anger externalizationwas not correlated with age (rho = 0.046, p = 0.502) as well asanger control rho = -0.005, p = 0.940;Fig. 1B, C).

### 4.2 Neural results

The Information Theoretic Criteria (Wax &Kailath, 1985) estimated eight networks of covarying grey matter (GM)and white matter (WM) that were subsequently estimated via tIVA. Each componentincluded an estimated GM component and a corresponding estimated WM componentwith a similar pattern of concentration across subjects. Positive valuesindicated increased concentration of GM/WM, while negative values indicated adecreased concentration. The loading coefficients of these eight components wereentered into two backward regression analyses, one to predict angerexternalization and one to predict anger control. For anger externalization, thefinal model was significant (R = 0.243, R^2^= 0.059,adjusted R^2^= 0.050, RMSE = 3.249, F = 6.557, p= 0.002), and included tIVA5 (beta = 52.000, p = 0.042),gender (beta = -1.161, p = 0.015), and the intercept (beta= 4.658, p = 0.245. tIVA5 indicates five out of eight networksestimated by the tIVA algorithm. The higher the externalization, the higher theGM–WM concentration inside this network. For anger control, the finalmodel was significant (R = 0.326, R^2^= 0.106, adjustedR^2^= 0.098, RMSE = 3.595, F = 12.435, p< 0.001), and included again tIVA5 (beta = -61.951, p =0.029), gender (beta = 2.021, p < 0.001), and the intercept (beta= 30.995, p < 0.001). The higher the control, the lower theGM–WM concentration inside this network. SeeTables 1and2fora description of the areas included in the network, andFigures 2and3, for avisual representation of GM and WM as well as the residual plots. To betterunderstand the direction of the effect of gender, we computed a t-test and wefound females having higher GM–WM concentration than men (t =3.287, p = 0.001). Although the regression did not return significanteffect of age, we additionally computed a correlation between tIVA5 and age, andwe found significant negative effect of age for tIVA5 (rho = -0.296, p< 0.001), meaning that the older the participants, the lesser theGM–WM concentration.

**Table 1.** tIVA5—GM results

Area	Brodmann area	Volume (cc)	Random effects: Max value (MNI, x, y, z)
Paracentral Lobule	4, 5, 6, 31	2.2/1.9	6.3 (0, -35, 53)/5.7 (3, -32, 53)
Superior Frontal Gyrus	6, 8, 9	1.0/1.1	6.2 (0, 11, 48)/6.0 (1, 8, 51)
Medial Frontal Gyrus	6, 8, 9, 10, 32	2.1/2.9	5.4 (-1, 11, 44)/5.5 (1, 25, 42)
Cingulate Gyrus	24, 32	1.0/1.0	5.0 (-1, 14, 41)/5.5 (1, 21, 40)
Precuneus	7	0.4/0.2	5.2 (0, -36, 47)/4.0 (3, -34, 45)
Extra-Nuclear	13	0.0/0.3	-999.0 (0, 0, 0)/4.2 (43, 11, -9)
Superior Temporal Gyrus	22, 38	0.1/0.6	3.7 (-40, 11, -12)/4.2 (45, 8, -7)
Anterior Cingulate	32	0.1/0.3	4.2 (0, 36, 20)/4.0 (3, 35, 23)
Inferior Frontal Gyrus	47	0.1/0.3	3.6 (-37, 11, -14)/4.1 (42, 14, -12)
Insula	13	0.1/0.3	3.5 (-43, 8, -5)/4.0 (45, 4, -5)
Sub-Gyral	∗	0.1/0.0	3.7 (-42, 11, -8)/-999.0 (0, 0, 0)

∗ Indicates that no Brodmann is provided.

**Table 2.** tIVA5—WM results

Area	Brodmann area	Volume (cc)	Random effects: Max value (MNI, x, y, z)
Middle Frontal Gyrus	6, 8, 9, 10	1.2/1.5	5.6 (-27, 41, 37)/5.1 (40, 49, 20)
Postcentral Gyrus	2, 3, 4, 5, 7	1.2/0.4	5.4 (-27, -43, 67)/4.7 (30, -32, 65)
Precentral Gyrus	4, 6, 9	1.0/0.3	5.1 (-33, -19, 59)/4.2 (15, -27, 64)
Inferior Parietal Lobule	40	0.4/0.6	4.4 (-48, -42, 39)/4.8 (52, -40, 39)
Sub-Gyral	∗	0.1/0.3	3.7 (-30, -45, 52)/4.8 (16, -25, 61)
Superior Frontal Gyrus	6, 8, 9, 10	0.8/0.6	4.6 (-24, 44, 36)/4.2 (43, 49, 22)
Superior Parietal Lobule	7	0.3/0.0	4.5 (-31, -45, 59)/-999.0 (0, 0, 0)
Uncus	38	0.1/0.0	4.2 (-24, 13, -30)/-999.0 (0, 0, 0)
Supramarginal Gyrus	∗	0.2/0.1	4.2 (-48, -40, 35)/3.6 (53, -38, 36)
Precuneus	19	0.2/0.0	4.1 (-30, -75, 40)/-999.0 (0, 0, 0)
Superior Temporal Gyrus	∗	0.2/0.0	3.9 (-24, 16, -32)/-999.0 (0, 0, 0)
Middle Occipital Gyrus	18	0.1/0.0	3.8 (-31, -91, 10)/-999.0 (0, 0, 0)
Middle Temporal Gyrus	21	0.1/0.1	3.7 (-40, 7, -32)/3.5 (56, -51, 5)
Cuneus	∗	0.1/0.0	3.7 (-13, -97, 9)/-999.0 (0, 0, 0)
Fusiform Gyrus	∗	0.1/0.0	3.6 (-49, -38, -22)/-999.0 (0, 0, 0)
Medial Frontal Gyrus	∗	0.0/0.1	-999.0 (0, 0, 0)/3.6 (16, 57, 0)

Note that significant WM portions are reported in this table byconsidering their adjacencies with GM regions. ∗ Indicatesthat no Brodmann is provided.

**Fig. 2.**
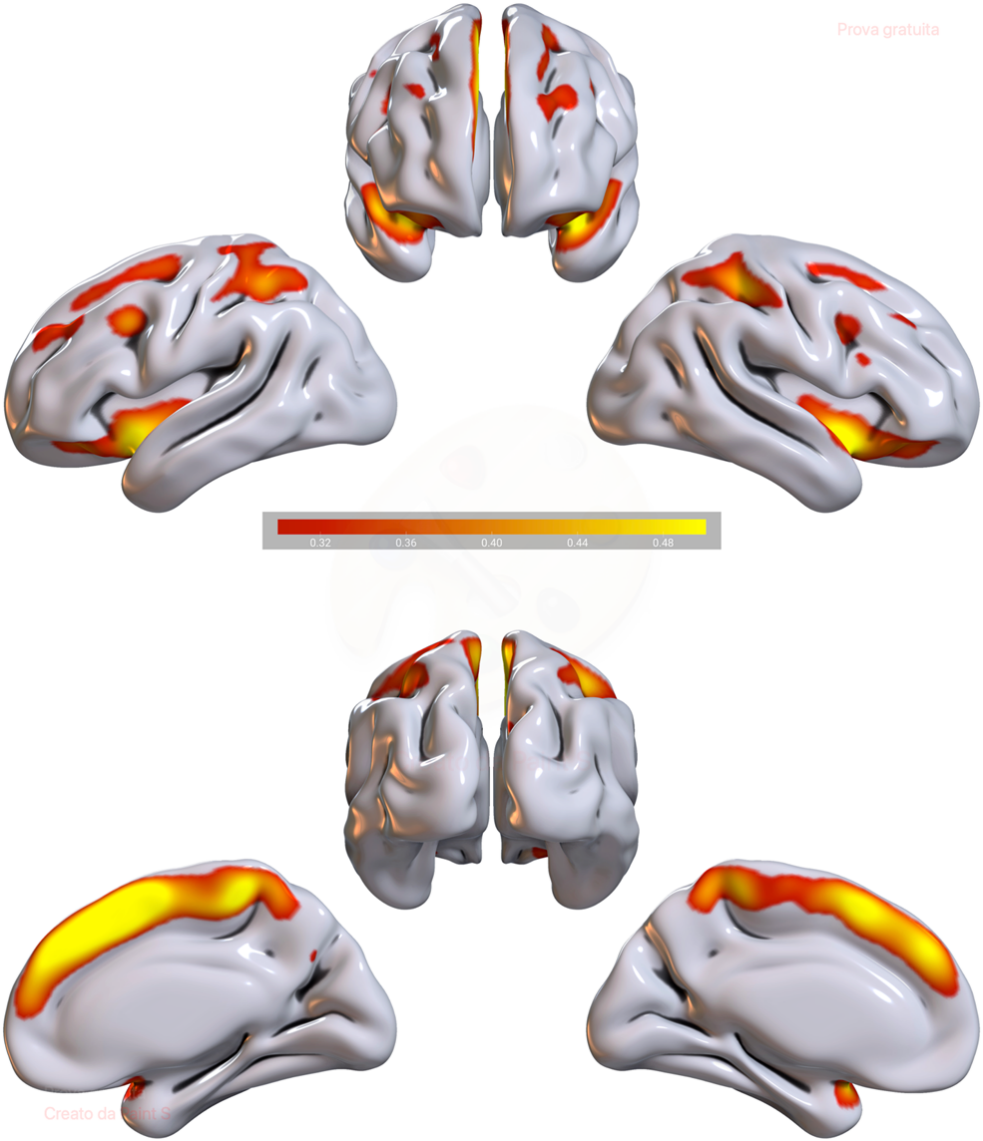
tIVA5 GM. Brain plots of tIVA5-GM. Regions showing increased GM and WMrepresented in warm colors.*Note*: the threshold was setat a z-score >2.

**Fig. 3.**
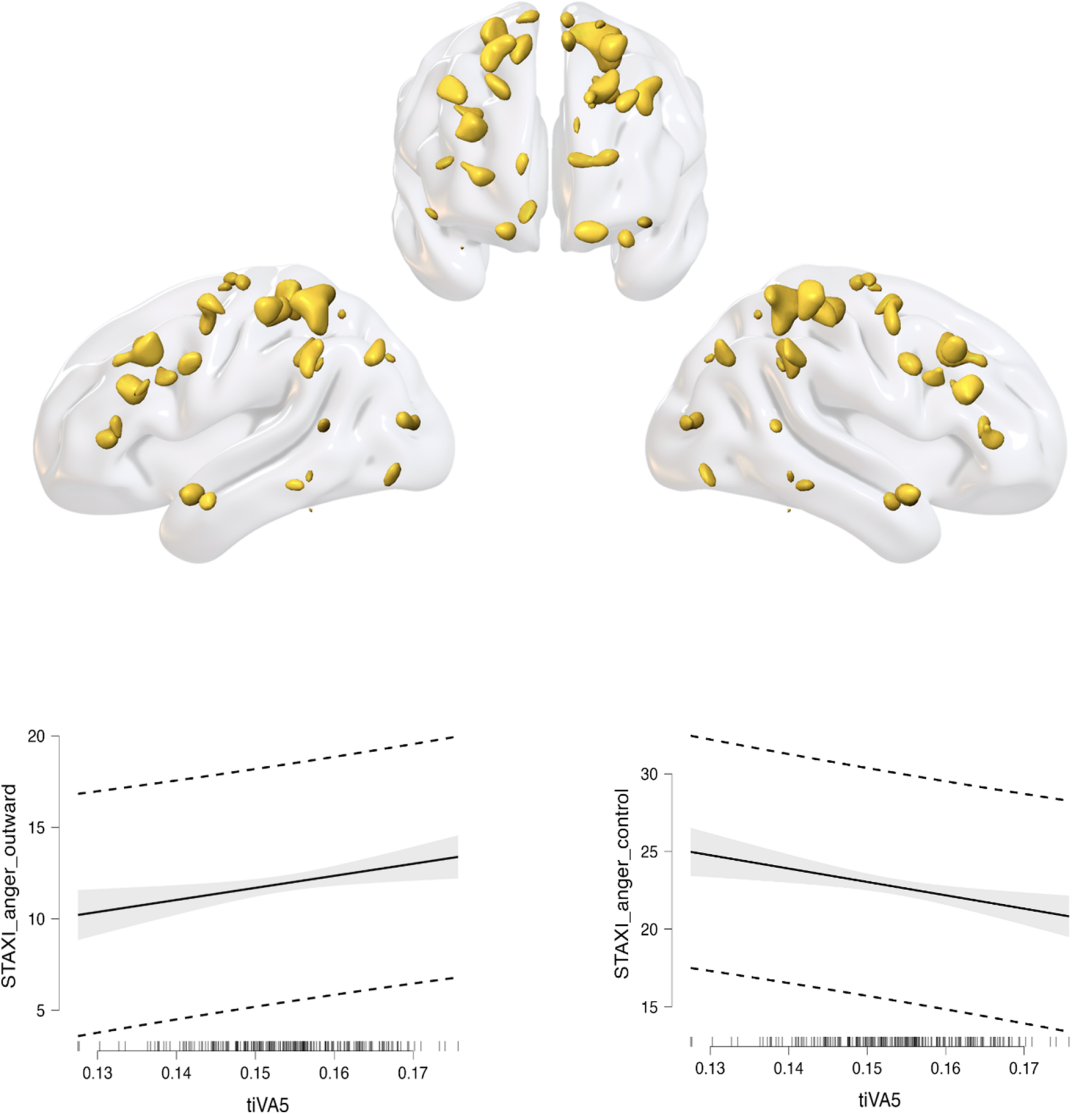
tIVA5 WM. 3D surface reconstruction plots of tIVA5–WM are shown.Note: the threshold was set at a z-score >2. The regressionresiduals plots, displayed at the bottom part of the figure, show thattIVA5 positively correlates with anger externalization and negativelywith anger control.

## 5 Discussion

The aim of this study was to test two hypotheses. The first hypothesis proposed anegative relationship between anger externalization and anger control. The secondhypothesis suggested that both the expression and control of anger could bepredicted, at the neural level, by the same GM–WM network. Specifically, afrontal control network may be involved in controlling and externalizing anger. Totest these hypotheses, we took into consideration behavioural scores from theSTAXI-2 subscales of anger out and anger control of 212 healthy participants, aswell as their GM and WM images. We found a significant negative correlation betweenanger externalization and control as predicted. Moreover, by applying anunsupervised machine learning method known as Transposed Independent VectorAnalysis, we found that one specific GM–WM network was able to predict boththe externalization and the control of anger. Departing from previous studies, weconsidered both GM and WM features in the same model. This allowed us to capturecomprehensive information about both aspects of brain structure, as certainpsychological processes rely on both tissue types (Baggio et al., 2023). In the following paragraphs, we provide details ofthese results.

At a behavioural level, we confirmed our hypothesis of a negative correlation betweenanger externalization and anger control. Thus, the individual tendency in directinganger outside (e.g., through aggressive behaviours) and the difficulty incontrolling, calming down, and monitoring the outcomes of anger are oftenconcomitant. It follows that anger externalization and control are twomanifestations of a common core difficulty in regulating anger. This result providesan empirical confirmation of a clinical intuition but also aligns with theIntegrative Cognitive Model (Wilkowski &Robinson, 2008,^2010^). Accordingto this model, controlling anger requires a form of effortful control, andindividual differences in providing such cognitive effort can importantly influenceanger externalization, mitigating aggression and reactivity in the presence of moreeffortful control resources. This model could also explain some aspects of angerdysregulation in many psychopathologies such as borderline personality disorder(De Panfilis et al., 2019) and antisocialpersonality disorder (Kolla et al., 2017),characterized by this combination of high anger externalization and low angercontrol.

Beyond self-report data, in the present study we provided, for the first time, aneurobiological explanation of this association, with the identification of aGM–WM circuit (tIVA5) that predicted both variables, with higherconcentrations associated with higher anger externalization and lower anger control.Conceptually, one can interpret this finding as a manifestation of a possible“anger regulation continuum” between externalization and control thatis reflected in the GM–WM features of this network. The fact that the samenetwork covaries with both anger facets may indicate that anger control andexternalization lie at the extremes of the same continuum. Higher concentration inthis anger regulation circuit corresponds on one extreme to high externalization/lowcontrol, whereas lower concentration reflects low externalization/high angercontrol. The placement of single individuals on this continuum can offer a usefulperspective for both clinical and research reflections concerning individual angermanagement, for the identification of potential tailored interventions, andassessment points.

The observation of the specific brain areas predicting individual differences inanger regulation may also shed light on psychological mechanisms that influence suchindividual differences. The most extended component of the circuit responsible forindividual differences in anger regulation was in supplemental motor areas (SMA) inthe paracentral lobule, with anterior extension towards the executive areas inmedial frontal gyrus and superior frontal gyrus. The importance of emotional motorcontrol, and motor planning have relevant implications for anger regulation (Friedman & Robbins, 2022). Consistently,the involvement of SMA may explain individual differences in anger regulation interms of differences in threshold for action. Coherently, a previous study foundthat trait anger modulates the brain connectivity between the bilateralsupplementary motor areas and the right frontal pole (Kim et al., 2022). The authors hypothesized that traitanger may be characterized by action readiness (supplementary motor area), moreinfluenced by self-referential and somatomotor information (hyperconnectivity withthe default mode and somatomotor networks). According toKohn et al. (2014), the SMA should be involved inexecution of regulation initiated by frontal areas, also detected in the presentstudy, and recognized in emotion regulation literature (for meta-analyses seeKohn et al., 2014;Messina et al., 2015).

For example, it has been affirmed that medial prefrontal regions should play a majorrole in anger regulation evaluating potential outcomes and directing behaviourstowards anger-eliciting stimuli (Gilam &Hendler, 2015). Interestingly, a previous study coherently found that theelectrical stimulation of the medial prefrontal cortex with transcranial directcurrent stimulation reduced anger reactions during an interpersonal game (Gilam et al., 2018). Finally, the involvementof prefrontal medial regions (including the cingulate) was found by a recentsupervised machine learning study to predict individual differences in angerexternalization from GM feature only (Grecucci etal., 2022).

Among prefrontal areas, also the inferior frontal gyrus has been detected in thepresent study. One recent meta-analysis bySorellaet al. (2021)showed that the right inferior frontal gyrus is active forboth the perception of angry stimuli and the subjective experience of anger. Thismay indicate that the higher the GM concentration in this circuit, the higher theanger experiences (and thus the externalization). From a functional point of view,anger externalization has been linked to brain connectivity in the prefrontalcortex, specifically between the inferior frontal gyrus and other cortical andsubcortical regions. This suggests a clear role modulating anger in the decision toexternalize it (Sorella & Grecucci,2023).

Another important region in our study was the cingulate cortex. The cingulate,especially the anterior part, as well as the medial prefrontal area, has beenassociated with angry rumination (Denson et al.,2009), which characterizes the internalization of anger (Consolini et al., 2022). Indeed, in one study, authorsfound that after the presentation of angry faces, the negative connectivity of theventral anterior cingulate cortex with the amygdala was reduced in individuals withhigh appetitive motivation (associated with aggression) (Passamonti et al., 2008). The involvement in the anteriorcingulate in rumination and internalization of anger may appear contradictory to ourfindings, which show a positive association between the cingulate andexternalization. However, in our study, the cingulate area was more centrallylocated and posterior compared with the anterior cingulate observed in previousstudies on rumination and internalization (Consoliniet al., 2022;Denson et al.,2009). In another functional study, after the presentation of angry faces,the negative connectivity of the anterior cingulate cortex with the amygdala wasaltered, especially in individuals with aggression (Passamonti et al., 2008). This study highlights a role of the cingulatein anger perception and in the modulation of other brain areas.

Another interesting region found inside the tIVA5 network was the precuneus. Althoughthe precuneus serves numerous functions, it is recognized to be the main hub of theposterior default mode network. In a recent study, it was found that anger controlwas associated with the default mode network (Sorella et al., 2022). The role of DMN in anger reaction/control couldbe responsible for hostile bias and reactivity in front of anger eliciting stimuli.In particular, the default mode network is responsible for self-referentialprocesses and rumination that are implicated in the experience of anger.

We also found a role for the insula. This is not surprising because the insula andother limbic and subcortical areas are all involved in the emotional experience(Picó-Pérez et al., 2018).For example, the insula has been found in angry reactions towards unfair behavioursin decision-making tasks (Grecucci et al.,2013a,2013b). Also, according toAlia-Klein et al. (2020), uncontrollableanger relies on a subcortical low road including the insula and the amygdala. Inanother study, it has been found that in response to prohibitive language,individuals with specific genotypes are more likely to experience anger, and thatthis relies on insula and right hippocampus activity (Alia-Klein et al., 2009). Moreover, the activity of theinsula is involved not only in anger and aggression (Dambacher et al., 2015;Emmerling etal., 2016;Skibsted et al., 2017),but also in anger rumination (Denson et al.,2009).

For what concerns the white matter side of the tIVA5 network, results indicate adiffuse effect of white matter portions adjacent to the relative main GM regionsdetected by the algorithm. Several WM portions have been found close to frontal andtemporal regions, thus supporting the functionality of the GM regions theycommunicate with. Of note, the method used in this study (tIVA) does not provideinformation on white matter fibres integrity as usually DTI does. WM was used in asimilar way as GM was, with the idea of detecting WM density and its associationwith anger externalization and control (seeBaggio etal., 2023;Grecucci, Sorella, et al.,2023;Grecucci et al., 2024;Jornkokgoud et al., 2024for similarmethods).

Another intriguing finding of the present study was that anger externalization andcontrol displayed were different in females and males, and coherently there is adifference in GM–WM concentration between females and males. In our sample,females were found to have higher externalization and coherently lower controlcompared with men. Coherently, the anger regulation circuit presented loadingcoefficients were higher in women than in men (higher GM–WM concentration infemales than in males). This result can be somewhat counter-intuitive because weknow from the literature that the tendency for men to aggress is more pronounced inman than in women (at least for aggression that produced pain or physical injury),whereas perceived aggressivity (e.g., perception that enacting a behaviour wouldproduce harm to the target, guilt, and anxiety in oneself) is higher in females(Bettencourt & Miller, 1996;Eagly & Steffen, 1986). There are somepossible explanations for why some women may experience higher levels of anger thanmen. One possibility is that women are more sensitive to social injustices, andexperience gender stereotypes, injustice, role conflicts, lack of decision-makingpower, and even harassment or abuse, and social and familial pressures (Thomas, 2005). Alternatively, it is possiblethat female participants in the present study are not representative of the generalpopulation. Finally, we should consider that self-reported anger expression/controldoes not coincide with behavioural manifestation of anger. In order to draw clearconclusions regarding gender differences in anger management and the related braincorrelates, future studies should investigate such differences on the basis ofobserved behaviours. The effect of gender was also visible in the regressionanalyses to predict both anger control and externalization. Indeed, additionalanalyses confirmed a clear effect of gender for tIVA5 network. Specifically, womenhad higher GM–WM concentration inside tIVA5 network. Since we found apositive correlation between tIVA5 and anger externalization (and negativecorrelation between tIVA5 and anger control), the fact that women had higherGM–WM concentration is coherent with the fact that women externalize angermore than men.

### 5.1 Translational implications

When anger is excessively externalized, individuals tend to express their angerthrough aggressive behaviours, which can lead to negative interpersonaloutcomes. Conversely, excessive suppression of anger is associated with adversetraits such as hypertension, low self-esteem, maladaptive thoughts andbehaviours, including rumination, and psychopathological conditions (Stimmel et al., 2005). Therefore, wesuggest that an optimal balance between the two is advisable. From atranslational perspective, intervening in the functionality of the GM–WMcircuit reported in this study may be an effective method to modulate excessesin both aspects of anger. Altering the functionality of this circuit in thedesired direction (e.g., reducing it in the case of excessive externalization orenhancing it in the case of excessive suppression) could be beneficial forindividuals with anger issues. Neurostimulation methods such as transcranialdirect current stimulation (tDCS) or transcranial magnetic stimulation (TMS) canbe valuable tools to achieve this goal. Personality disorders characterized bydifficulties in anger regulation may be particularly good candidates for suchinterventions, especially borderline personalities (De Panfilis et al., 2019;Grecucci et al., 2022;Grecucci, Dadomo, et al., 2023), and antisocialpersonalities (Kolla et al., 2017).Beside clinical implications, the combination of neuromodulation techniques withfunctional magnetic resonance imaging can provide causal information on the roleof the brain circuit found in this study in modulating behavioural outcomes ofanger externalization and control.

## 6 Conclusions and Limitations

This study expands on previous research on the neural bases of anger, by adding newevidence that a network of covarying GM–WM predicted anger externalizationand control. These findings may help future treatment of people suffering from angerdysregulation. Several psychopathologies are associated with excessive angerexternalization or lack of control, including borderline personality disorder,antisocial personality disorder, intermittent explosive disorder, and anxietydisorders (Coccaro et al., 2014;De Panfilis et al., 2019;Kolla et al., 2017). However, excessive anger controlcharacterizes anxiety disorders and dependent personality disorder (Grecucci et al., 2020). In this context, this studyreveals potential clinical value, as it could be used for diagnostic or predictivepurposes regarding the onset of anger-related disorders. This circuit could beconsidered a potential target of neurostimulation treatment to reduce angerexternalization and increase anger control.

This study does not come without limitations. We investigated individual differencesin anger regulation on the basis of self-reported questionnaires. Although STAXI isa widely recognized questionnaire whose reliability has been confirmed by severalindependent studies (Deffenbacher et al.,1996;Foley et al., 2002;Forgays et al., 1997;Fuqua et al., 1991;Spielberger, 1996;Tibubos et al.,2020), it must be acknowledged that cultural differences may exist in howwe express and control anger across different populations (Alcázar Olán et al., 2015). Our resultsmay, therefore, be limited to the European culture, and future studies may want toconfirm these results across other cultures.

Another limitation is that this study focused only on structural analysis, leaving uswithout knowledge of activation patterns related to anger externalization/control.Future studies may want to explore the possibility of fusing structural andfunctional MRI data to expand these results. While further research is necessary, webelieve that these findings could provide valuable insights for understanding and inthe future predicting anger-related problems. The available data could ultimatelyassist healthcare practitioners in developing specific psychological treatments,such as targeted interventions involving brain stimulation/modulation orpharmacological techniques, for individuals experiencing anger-related issues.

## Data Availability

Behavioural and structural MRI data were selected from the open-access MPI-LeipzigMind Brain-Body dataset (OpenNeuro Dataset,http://openneuro.org, RRID:SCR_005031, accession number ds000221).
